# Supplementary figures and images for: Denuded Descemet’s membrane supports human embryonic stem cell-derived retinal pigment epithelial cell culture
Source: PLoS One. 2023 Feb 6;18(2):e0281404. doi: 10.1371/journal.pone.0281404 (PMC9901769; doi:10.1371/journal.pone.0281404)

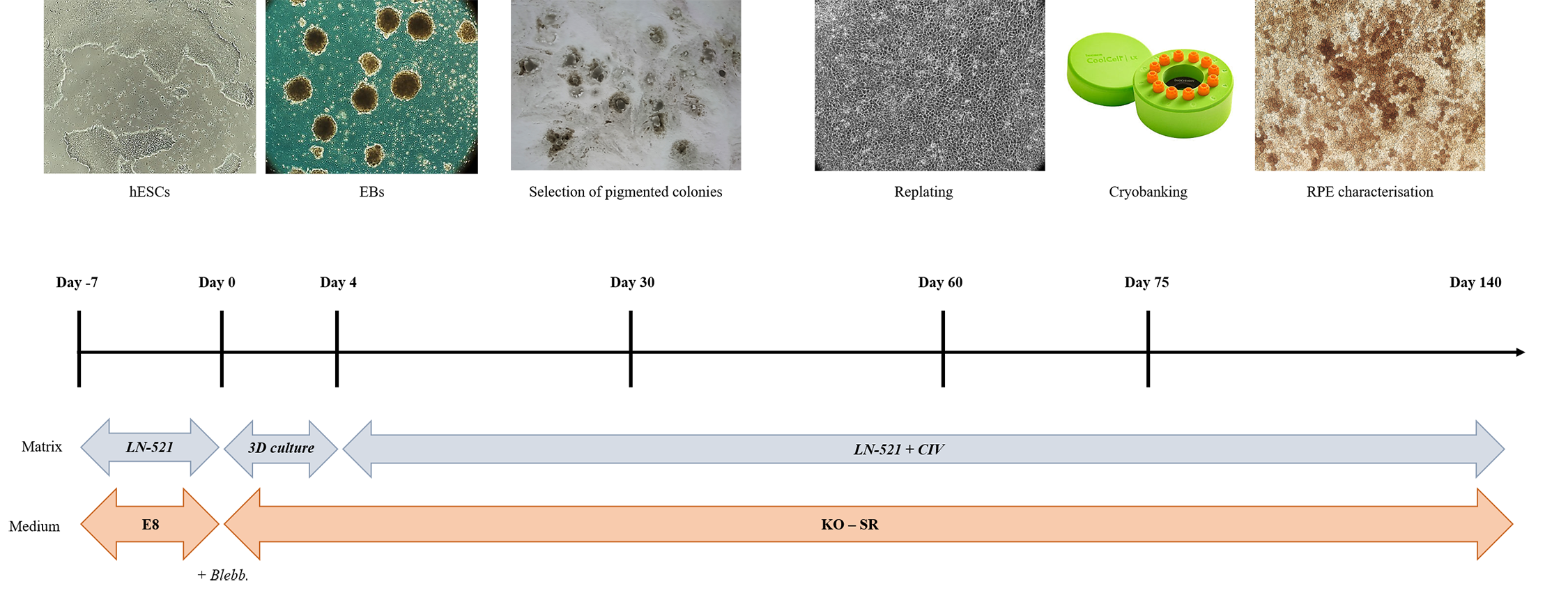

Supplement: S1 Fig — hESC, human embryonic stem cell; EB, embryoid bodies; RPE, retinal pigment epithelium; LN-521, recombinant laminin-521; CIV, collagen type IV; E8, Essential 8™ Flex Medium; Blebb, blebbistatin; KO-SR, Knock-out™ serum replacement. (TIF) [file pone.0281404.s001.tif]

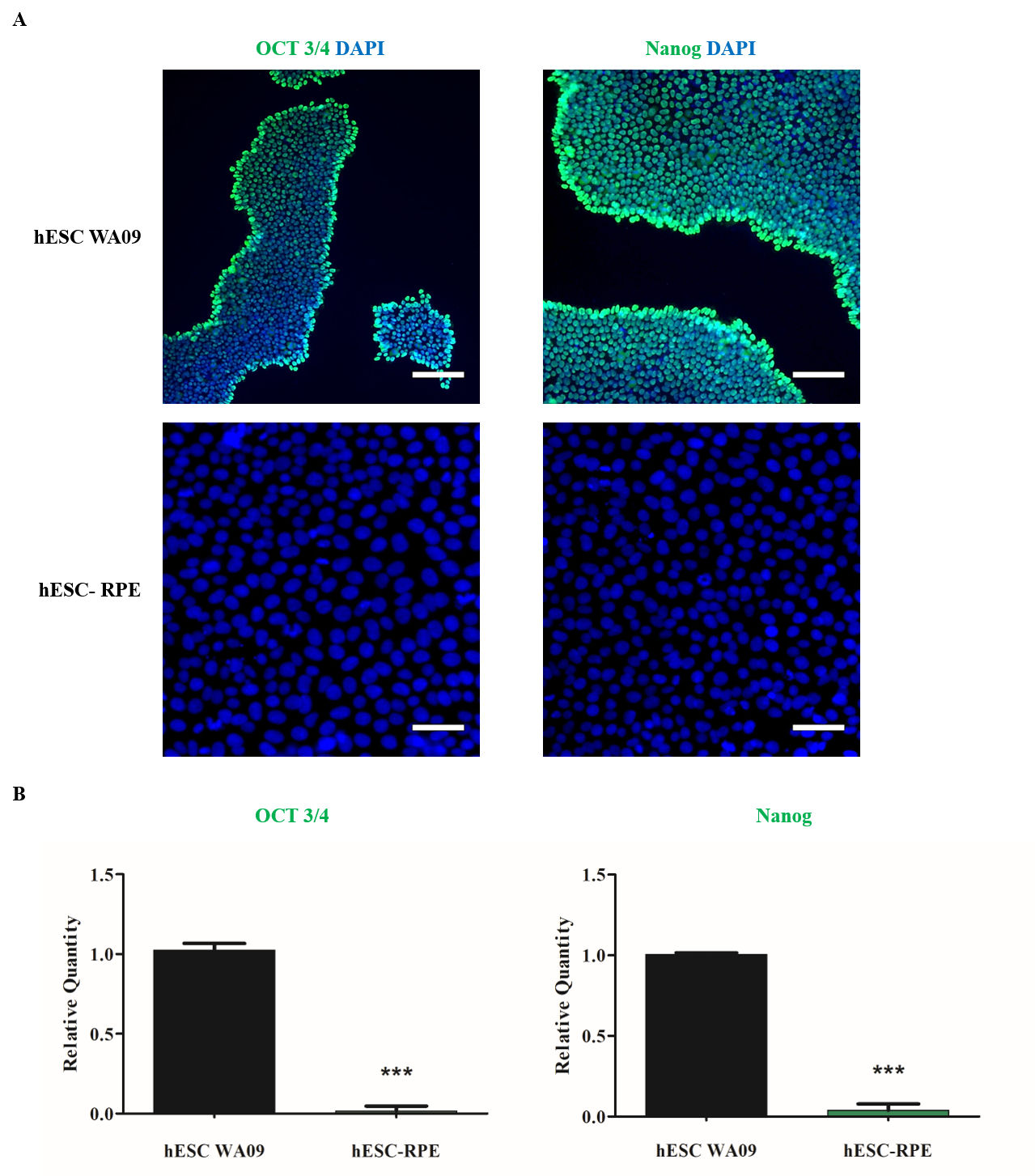

Supplement: S2 Fig — (A) Immunofluorescence showing lack of staining for pluripotency associated factors such as OCT-3/4 and Nanog (all green) in hESC derived-RPE cells (upper panel). hESC WA09 cell line was used for comparison (lower panel). Cell nuclei were stained with DAPI (blue). Scale bars = 30 μm. (B) Quantitative polymerase chain reaction revealed no expression of OCT-3/4 and Nanog in hESC-RPE. Data are normalized to hESC WA09 cell line. Data represent mean ± SD of N = 3 experiments for each sample. *** p<0.0001. hESC, human embryonic stem cell; RPE, retinal pigment epithelium. (TIF) [file pone.0281404.s002.tif]

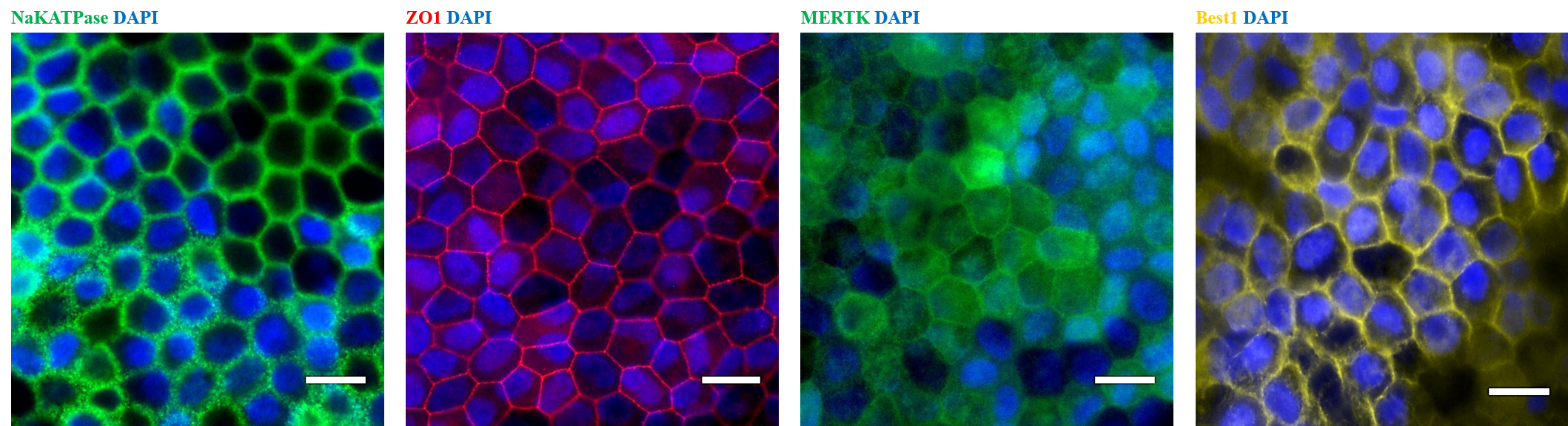

Supplement: S3 Fig — Representative immunofluorescence staining indicating the correct expression and localization of typical RPE markers in hESC-RPE cells merged with Hoechst nuclear staining (blue). Scale bars = 30 μm. Best1, Bestrophin 1; ZO1, zona occludens-1. (TIF) [file pone.0281404.s003.tif]

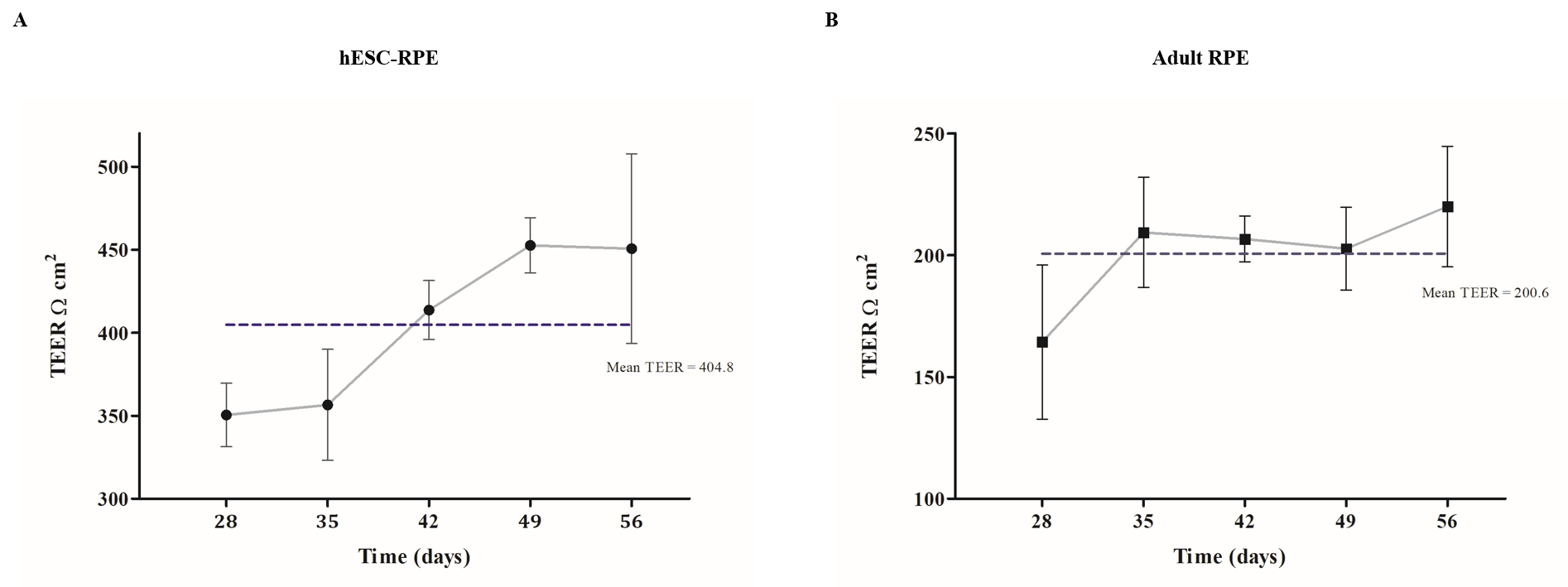

Supplement: S4 Fig — TER was measured to monitor the barrier function of hESC-RPE cells (A) and adult RPE cells (B) on TC inserts over time (days) using a Millicell volt/ohm meter. Data represent mean ± SD of N = 3 experiments for each sample. TER, transepithelial electrical resistance. (TIF) [file pone.0281404.s004.tif]

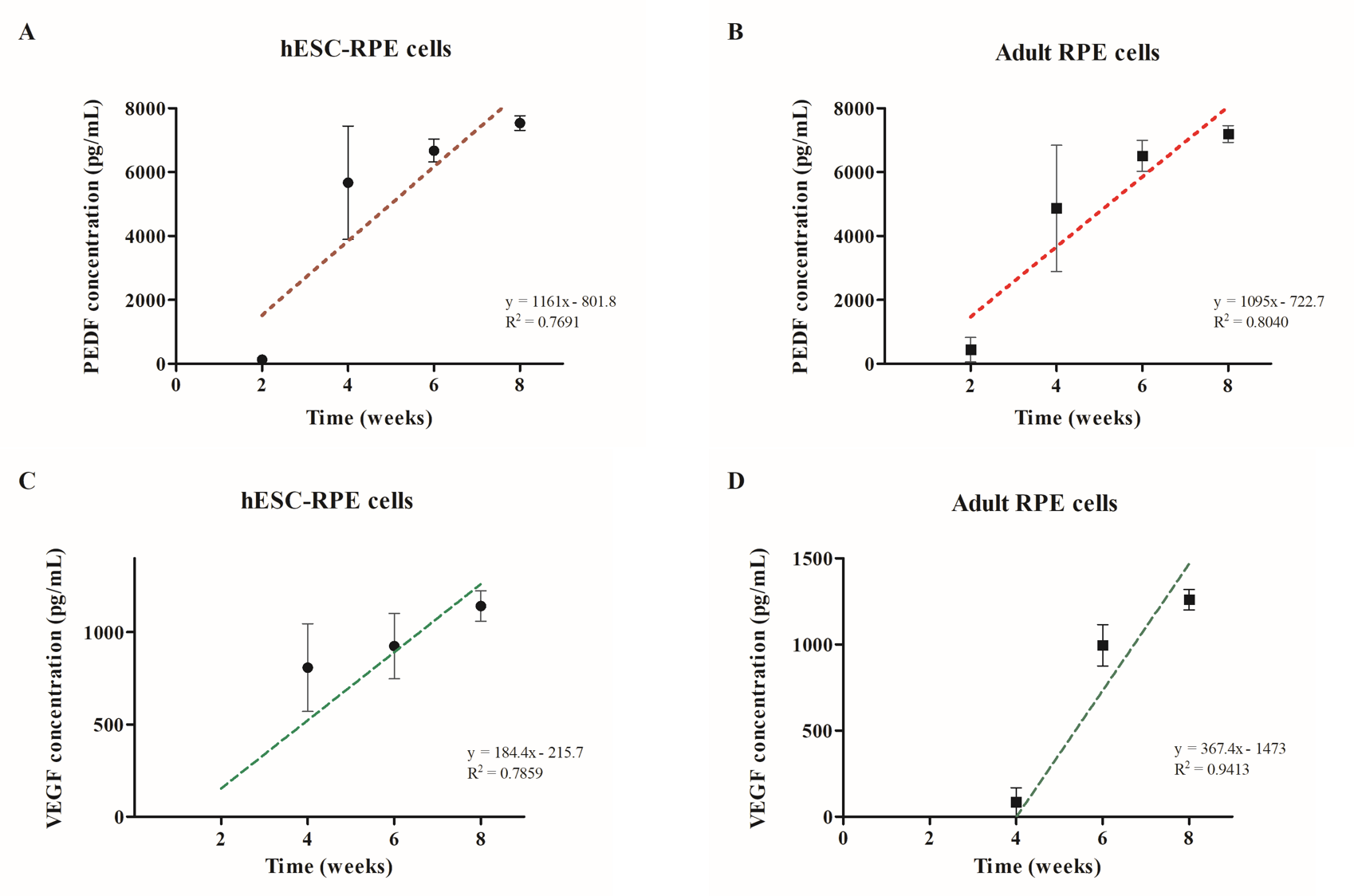

Supplement: S5 Fig — Spent culture media were collected from the apical and the basal chambers of the TC inserts and quantified for PEDF (A-B) and VEGF (C-D) concentration using ELISA assays in hESC-RPE cells (A, C) and adult RPE cells (B, D), respectively. Data represent mean ± SD of N = 3 experiments for each sample. (TIF) [file pone.0281404.s005.tif]

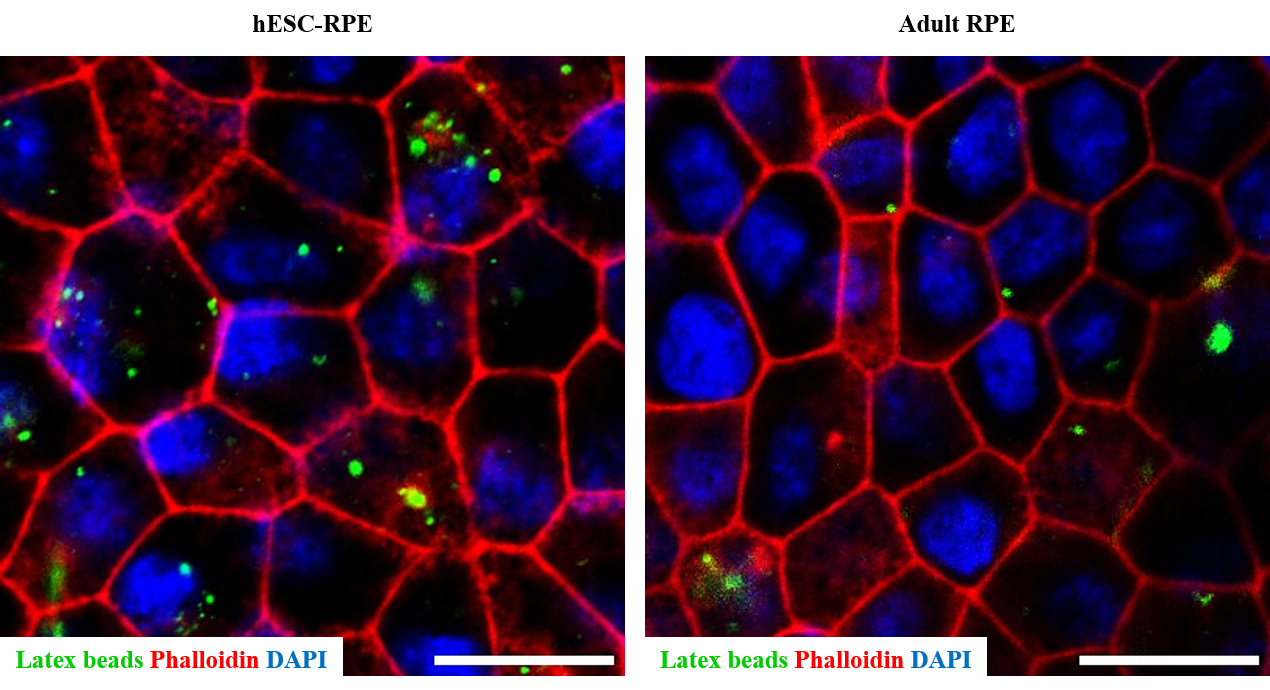

Supplement: S6 Fig — Phagocytic function of hESC-RPE cells and adult RPE cells was tested in vitro by challenging the cells with FITC-labeled latex beads. Beads ingestion (green) was observed for hESC-RPE cells, as well as for adult RPE cells. Phalloidin staining (red) was used to enhance the polygonal shape of the cells. Nuclei were counterstained with Hoechst (blue). Scale bars = 25 μm. (TIF) [file pone.0281404.s006.tif]
