# Supplementary material for: Denuded Descemet’s membrane supports human embryonic stem cell-derived retinal pigment epithelial cell culture
Source: PLoS One. 2023 Feb 6;18(2):e0281404. doi: 10.1371/journal.pone.0281404 (PMC9901769; doi:10.1371/journal.pone.0281404)
Supplement: S1 Appendix — (DOCX) [file pone.0281404.s007.docx]

**S1 APPENDIX - CHARACTERIZATION OF hESC-RPE CELLS**

**S1 APPENDIX - METHODS**

**PURITY OF hESC-RPE CELLS**

Immunohistochemistry and quantitative real-time polymerase chain reaction (Q RT-PCR) were used to confirm the purity of RPE cells culture (Figure A2). Undifferentiated hESC WA09 cell line was taken as positive control. For gene expression analysis, total RNA was extracted with miRNeasy Tissue/Cells Advanced Mini Kit (Qiagen) following manufacturer’s instructions and RNA concentration was calculated with Qubit 2.0 Fluorometer (Thermo Fisher Scientific). cDNA was synthesized using the High Capacity cDNA Reverse Transcription Kit (Thermo Fisher Scientific), according to the kit protocol. Q RT-PCR was performed with TaqMan Universal Matermix and predesigned TaqMan assays with FAM-labels (all from Thermo Fisher Scientific). The reactions were carried out on ABI Prism 7900HT Sequence Detection System (Applied Biosystems). Relative gene quantification was measured by using the 2^-ΔΔCt^ method. GAPDH was the internal housekeeping gene reference for normalization. Stem cell markers OCT-3/4 and Nanog were not expressed in derived RPE cells, while high expression levels could be detected in hESC WA09 cell line. Immunofluorescence was carried out using the protocol described in the paragraph below.

**IMMUNOFLUORESCENCE**

hESC WA09 and hESC-RPE cells were washed with PBS and fixed in 4% paraformaldehyde (Santa Cruz Biotechnology) for 15 minutes at room temperature (RT). After several PBS washings, the cells were permeabilized with 0.1% Triton X-100 (Sigma-Aldrich) in PBS for 15 minutes at RT. Cells were washed with PBS and blocked with 3% BSA (Sigma-Aldrich) in PBS at RT for 1 hour to minimize unspecific binding sites. Samples were incubated at 4°C overnight with primary antibodies diluted in 3% BSA at the specified concentrations: Bestrophin 1:100 (PA589606), zonula occludens 1 (ZO-1) 1:200 (61-7300) (both from Thermo Fisher Scientific), Na+/K+ ATPase 1:200 (ab7671, Abcam), MERTK 1:50 (H00010461-M01, Abnova), OCT-3/4 1:200 (AF1759) and Nanog 1:150 (AF1997) (both from R&D Systems). The following day cells were washed with PBS and secondary antibodies were added at a dilution of 1:200 in 3% BSA for 1 hour at RT. Alexa Fluor 594-conjugated goat anti-mouse IgG (A11032), 594 donkey anti-rabbit IgG (A21207), 488-conjugated donkey anti-mouse IgG (A21202), A488 goat anti-rabbit IgG (A11034) and 488 donkey anti-goat IgG (A11055) were used as secondary antibodies (all from Thermo Fisher Scientific). Nuclei were counterstained with the nuclear dye Hoescht diluted 1:3000 in PBS at RT for 5 minutes. The samples were mounted in ProLong Gold Antifade Mountant without DAPI (Thermo Fisher Scientific). Images were captured with Nikon fluorescent microscope (Nikon) using 40x oil immersion objective (Figure A3).

**TRANSEPITHELIAL RESISTANCE (TER)**

Transepithelial electrical resistance (TER) was measured for hESC-RPE cells as well as for adult RPE cells cultured on TC inserts with the Millicell® ERS-2 meter and electrode system (Merck Millipore) at days 28, 35,42,49 and 56 (Figure A4). To obtain true resistance value, resistance measured across the samples was subtracted from resistance read across the controls, respectively denuded hAM and TC insert without cells. Finally, TER (Ω cm^2^) measure was acquired by normalising the resistance value with the surface area of the nitinol ring or the TC insert.

**ENZIME-LINKED IMMUNOSORBENT ASSAY**

hESC-RPE cell and adult RPE cell culture supernatants from the upper and lower compartments of the TC inserts were collected after 48 hours of cell culture at 37°C. Harvested media were diluted 1:2 and apical PEDF secretion levels were analysed with Human SERPIN F1 ELISA kit (Thermo Fisher Scientific). Basal media were diluted 1:10 for VEGF quantification using Human VEGF ELISA kit (Thermo Fisher Scientific), following manufacturer’s instructions (Figure A5). Media volume and insert growth area were used to calculate PEDF and VEGF levels.

**PHAGOCYTOSIS ASSAY**

Mature hESC-RPE cells and adult RPE cells cultured on TC inserts for 56 days were incubated with FITC-labeled latex beads (Sigma-Aldrich) for 48 hours at 37°C in RPE cell culture medium. After this time, cells were washed three times in PBS and then fixed with 4% paraformaldehyde for 15 minutes at RT following PBS washings. Cells were permeabilized for 15 minutes with 0.1% Triton X-100 in PBS at RT followed by PBS washings. The blocking step was performed by incubation in 3% (w/v) BSA antibody diluent for 1 hour at RT. The cells were washed repeatedly in PBS and then labelled with 1:1000 phalloidin (Sigma P1951) in 3% BSA in PBS for 1 h at RT. Cell nuclei were counterstained with Hoechst (Thermo Fisher Scientific) diluted 1:3000 in PBS for 5 minutes at RT, following repeated PBS washings. The cells were finally mounted with ProLong Gold Antifade Mountant without DAPI (Thermo Fisher Scientific) and imaged using Nikon Eclipse Ti-E fluorescent microscope (Nikon).
