# Supplementary material for: Denuded Descemet’s membrane supports human embryonic stem cell-derived retinal pigment epithelial cell culture
Source: PLoS One. 2023 Feb 6;18(2):e0281404. doi: 10.1371/journal.pone.0281404 (PMC9901769; doi:10.1371/journal.pone.0281404)
Supplement: S2 Appendix — (PDF) [file pone.0281404.s008.pdf]

School of Medicine and Public Health
UNIVERSITY OF WISCONSIN-MADISON
Department of Pathology and Laboratory Medicine
TRIP Laboratory (Molecular)
http://www.pathology.wisc.edu/research/trip

Short Tandem Repeat Analysis

WiCell®
info@wicell.org
(888) 204-1782

Sample Report:
12726-STR
Sample Name on Tube: 12726-STR
57.4 ng/μL, (A260/280=1.96)
Sample Type: Cells
Cell Count: ~2 million cells

Requestor:
WiCell Research Institute
Quality Department

Sample Date: N/A
Receive Date: 08/14/17
Assay Date: 08/16/17
File Name: STR 170817 wmr
Report Date: 08/21/17

STR Locus	STR Genotype Repeat #	STR Genotype
**FGA**	16–18,18.2,19,19.2,20,20.2,21,21.2,22, 22.2, 23, 23.2, 24, 24.2, 25, 25.2, 26–30, 31.2, 43.2, 44.2,45.2, 46.2	26,28
**TPOX**	6-13	10,11
**D8S1179**	7-18	8,14
**vWA**	10-22	17,17
**Amelogenin**	X,Y	X,X
**Penta_D**	2.2, 3.2, 5, 7-17	9,13
**CSF1PO**	6-15	11,11
**D16S539**	5, 8-15	12,13
**D7S820**	6-14	9,11
**D13S317**	7-15	9,9
**D5S818**	7-16	11,12
**Penta_E**	5-24	11,14
**D18S51**	8-10, 10.2, 11-13, 13.2, 14-27	13,13
**D21S11**	24,24.2,25,25.2,26-28,28.2,29,29.2, 30, 30.2,31, 31.2,32,32.2,33,33.2, 34,34.2,35,35.2,36-38	30,30
**TH01**	4-9,9.3,10-11,13.3	9.3,9.3
**D3S1358**	12-20	13,16

Results: Based on the 12726-STR cells submitted by WiCell QA dated and received on 08/14/17, this sample (Label on Tube: 12726-STR) exactly matches the STR profile of the human stem cell line WA09 comprising 24allelic polymorphisms across the 15 STR loci analyzed.

Interpretation: No STR polymorphisms other than those corresponding to the human WA09 stem cell line were detected and the concentration of DNA required to achieve an acceptable STR genotype (signal/ noise) was equivalent to that required for the standard procedure (~1 ng/amplification reaction) from human genomic DNA. This result suggests that the 12726-STR sample submitted corresponds to the WA09 stem cell line and was not contaminated with any other human stem cells or a significant amount of mouse feeder layer cells.

Sensitivity: Sensitivity limits for detection of STR polymorphisms unique to either this or other human stem cell lines is ~2-5%.

X *RMB* **Digitally Signed on 08/23/17**

[redacted]
TRIP Laboratory, Molecular

X *WMR* **Digitally Signed on 08/23/17**

[redacted], *PhD, Director / Co-Director*
UWHC Molecular Diagnostics Laboratory / UWSMPH TRIP Laboratory

Testing was accomplished by analysis of human genetic polymorphisms at STR loci. This methodology has not yet been approved by the FDA and is for investigational use only.
Acknowledge TRIP in your publications, posters & presentations. For details, see: *http://www.pathology.wisc.edu/research/trip/acknowledging*
TRIP agrees to maintain the confidentiality of any information provided to it in connection with its performance of this STR analysis on the same conditions as set forth in paragraph 2 of WiCell's Terms and Conditions of Service (http://www.wicell.org/media.acux/1a429b84-2b54-44a4-8ad8-5c05db93dd8a).
