# Supplementary material for: Denuded Descemet’s membrane supports human embryonic stem cell-derived retinal pigment epithelial cell culture
Source: PLoS One. 2023 Feb 6;18(2):e0281404. doi: 10.1371/journal.pone.0281404 (PMC9901769; doi:10.1371/journal.pone.0281404)
Supplement: Attachment — Submitted filename: PONE-D-22-26643 _review.docx [file pone.0281404.s009.docx]

The purpose of this study was to investigate whether decellularized Descemet’s membrane (DM) supports the maturation of hESC-derived RPE cells. DMs were isolated from human corneas, corneal endothelium was peeled off and additional Thermolysin treatment was applied. Decellularization was confirmed by immunohistochemistry and Atomic Force Microscopy (AFM). dDM was dried out on tissue culture plates or PET tissue culture inserts and sterilized by UV. hESC-derived RPE cells attached faster and showed higher initial proliferation when plated on the corneal endothelial side of acellular DM (dDM). RPE on dDM produced significantly more VEGF at 4 weeks than RPE cultured on tissue culture inserts. The transepithelial resistance was lower in RPE cultures on dDM, but reached an equal value on d56. hESC-RPE cultures on dDM were polarized and expressed significantly more RPE65. As a control, the authors also cultured adult eyebank-derived RPE cells which similarly showed a higher dree of maturation and significantly more RPE65 expression on dDM. Thus, the authors conclude that dDM is a suitable substrate for RPE cells.

General comments: The paper is well written, and the studies are well designed. The methods are well described, with listing experimental numbers. The figures are excellent. The results are important because dDM is a natural substrate for RPE cells, could serve as a xeno-free substrate for hESC-RPE, and could help to improve transplantation outcomes.

The authors also discuss the limitations of their study as they have not yet tried to transplant RPE sheets on dDM. It is unclear whether dDM has the same diffusion properties as Bruch’s membrane. This is an important question that needs to be answered because of the RPE role in transporting nutrients for photoreceptors from the choroid.

Specific comments:

A data availability statement is missing from the manuscript and only entered in the manuscript submission form. The authors state that some restrictions will apply, but do not specify which restrictions. The authors state that all relevant data are withing the manuscript and its supporting information files.

Author contributions: The authors should list the specific contributions for each author.

(Minor):

p.14, line 340: “showed” should be “shown”
